# Supplementary material for: Identification and engineering of highly functional potyviral proteases in cells using co-evolutionary models
Source: Nat Commun. 2026 Feb 26;17:3257. doi: 10.1038/s41467-026-69961-5 (PMC13062108; doi:10.1038/s41467-026-69961-5)
Supplement: Supplementary file 1 — Supplementary Information [file 41467_2026_69961_MOESM1_ESM.pdf]

Supplementary Information for "Identification and engineering of highly functional Potyviral proteases in cells using co-evolutionary models"

Medel B. Lim Suan Jr.[1†], Cheyenne Ziegler[2†], Zain Syed[2], Arjun Sai Yedavalli[2], Ajay Tunikipati[2], Rodrigo Raposo[2], Jaimahesh Nagineni[2], Jaideep Kaur[1], Faruck Morcos[1,2,3,4*], and P. C. Dave P. Dingal[1,2,3*]

[1]Department of Bioengineering, [2]Department of Biological Sciences, [3]Center for Systems Biology, [4]Department of Physics, UT Dallas, 800 N Loop Rd, Richardson, TX 75080

[†] These authors contributed equally.
[*] Corresponding authors

Table of Contents

Supplementary Figures

Supplementary Figure 1: Normalized cleavage-induced fluorescence of 31 Potyviral proteases against their 7-residue substrates.

Supplementary Figure 2: Experimental validation of orthogonality of 225 protease-substrate pairs.

Supplementary Figure 3: Comparative receiver operating characteristic (ROC) analysis of raw and normalized H_{spec} predictors.

Supplementary Figure 4: Proteolytic cleavage activity of mutants with similar H_{spec} to wildtype counterparts.

Supplementary Figure 5: Predicted and experimentally measured proteolytic cleavage of cognate and point-mutant substrates.

Supplementary Figure 6: ProSSpeC can prescribe single-site mutations that elicit crosstalk between protease and substrate that do not originally interact.

Supplementary Figure 7: Top 50 most negative DeltaH of P2-coupled EAPVp residues between EAPVp-SPV2cs and EAPVp-mutSPV2cs.

Supplementary Figure 8: Gating strategy for selective synpoptosis of mutant cells in a mixed cell population.

Supplementary Figure 9: Orthogonality matrix of normalized masked H_{spec} scores.

Supplementary Figure 10: Sequence logo of NIa-protease substrates used for concatenated alignment.

Supplementary Figure 11: Top direct information (DI) contacts in the monomeric structure of TEVp.

Supplementary Figure 12: Interfacial DI contacts between TEVp and its 7-amino-acid substrate.

Supplementary Figure 13: Comparison between fluorescence microscopy and flow cytometry for quantifying cleavage-induced fluorescence.

Supplementary Figure 14: Protease titration.

Supplementary Tables

Supplementary Table 1: Equivalent positions of TEVp(S219V).

Supplementary Table 2: Masked versus Full H_{spec} for all natural proteases tested against their cognate 7-residue substrates.

Supplementary Table 3: Predicted Δ H_{spec} (DeltaH) and corresponding change in fluorescence (DeltaFluor) between the sample pair and the reference pair.

Supplementary Table 4: *Potyviridae* sequences utilized in this study.

Supplementary Table 5: Gated flow cytometry cell counts capture protease-induced, caspase-mediated apoptosis (synpoptosis).

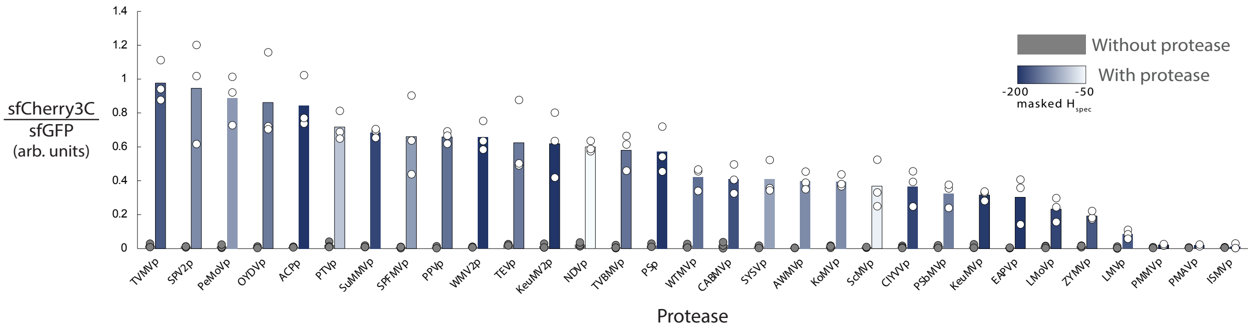

Supplementary Figure 1: Normalized cleavage-induced fluorescence of 31 Potyviral proteases against their 7-residue substrates. Bars represent mean values from 3 independent replicates. Bars are shaded based on the masked H_{spec} heatmap. Values at 0.05 a.u. or lower indicate no cleavage-induced fluorescence, as they are barely detected above background noise. Arb. units, arbitrary units. Source data are provided as a Source Data file.

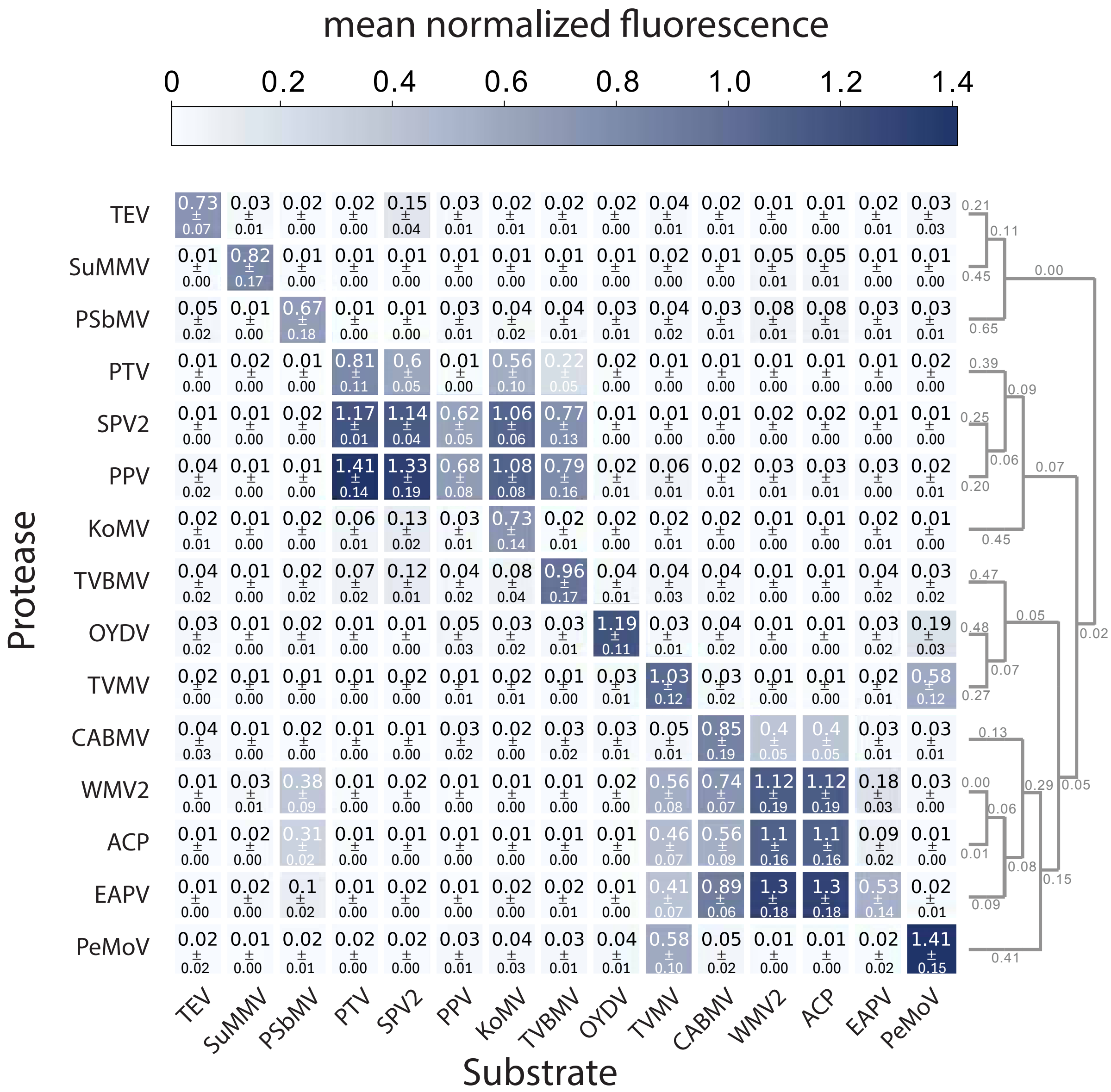

Supplementary Figure 2: Experimental validation of orthogonality of 225 protease-substrate pairs. Values are means of normalized cleavage-induced fluorescence (sfCherry3C/sfGFP) ± SEM, derived from 3-5 independent replicates. Phylogenetic tree is the same as in Fig. 3b. Source data are provided as a Source Data file.

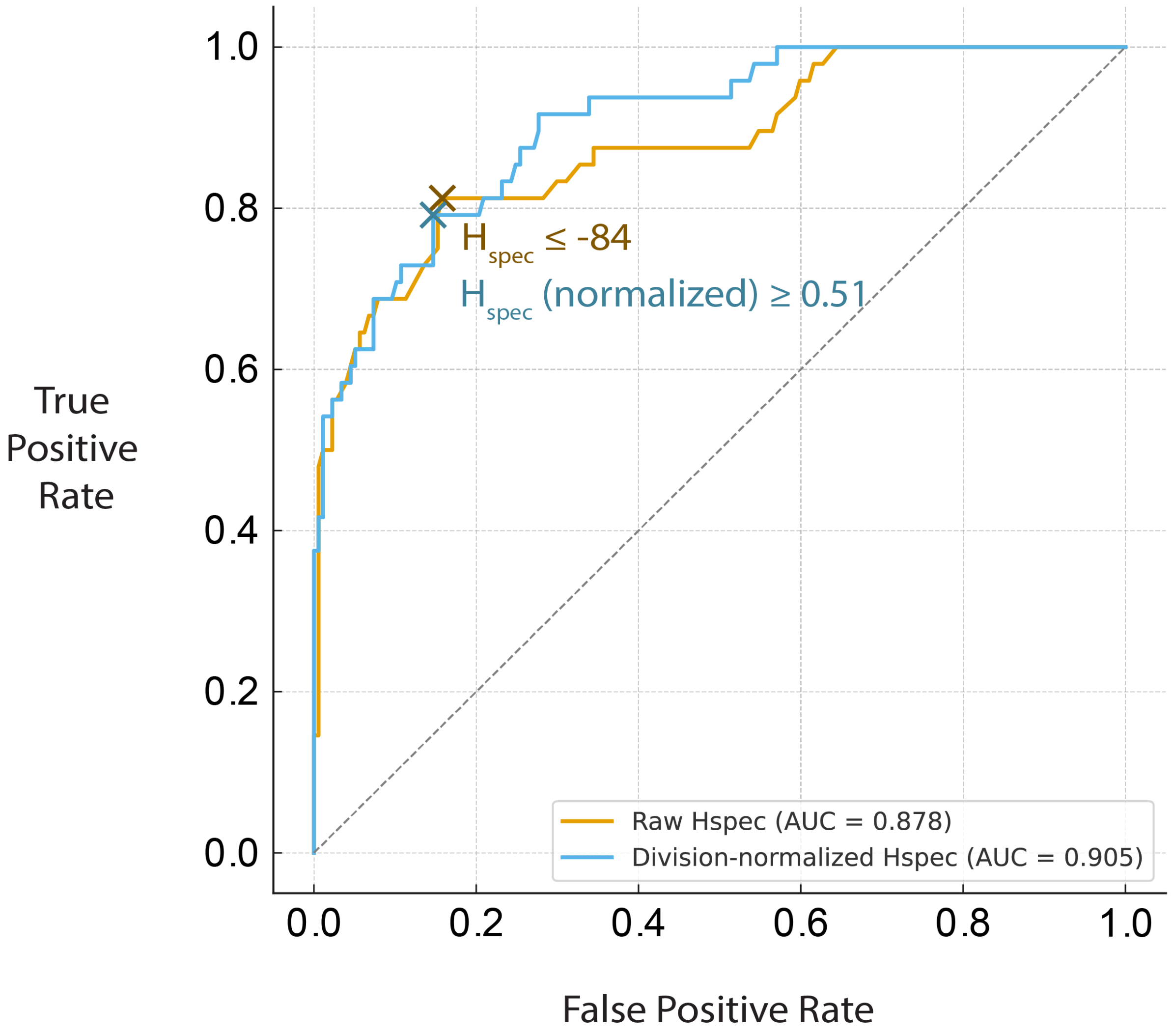

Supplementary Figure 3: Comparative receiver operating characteristic (ROC) analysis of raw and normalized H_{spec} predictors. ROC curves showing the ability of the theoretical H_{spec} score to discriminate cleaving (fluorescence ≥ 0.1) from non-cleaving protease–substrate pairs. True positive rate and false positive rate were calculated using Equations 6 & 7, respectively. H_{spec} normalization improved predictive performance relative to the raw H_{spec}, yielding a higher area under the curve (AUC ≈ 0.91). The dashed diagonal line denotes random classification. Source data are provided as a Source Data file.

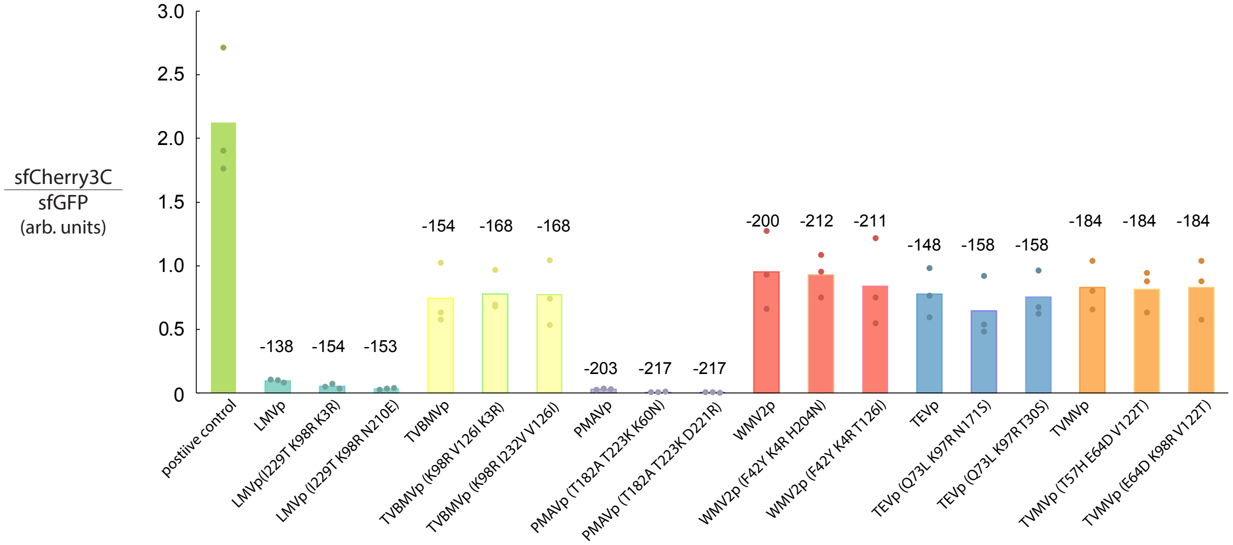

Supplementary Figure 4: Proteolytic cleavage activity of mutants with similar H_{spec} to wildtype counterparts. For each protease, two mutants were engineered and tested (bars matching wildtype color). Bars depict cleavage-induced fluorescence for each protease. H_{spec} scores are shown above the bars. Positive control represents cytoplasmic free-floating sfCherry3C(1-10) with nuclear reporter. Bars represent the means of 3 independent experiments. Arb. units, arbitrary units. Source data are provided as a Source Data file.

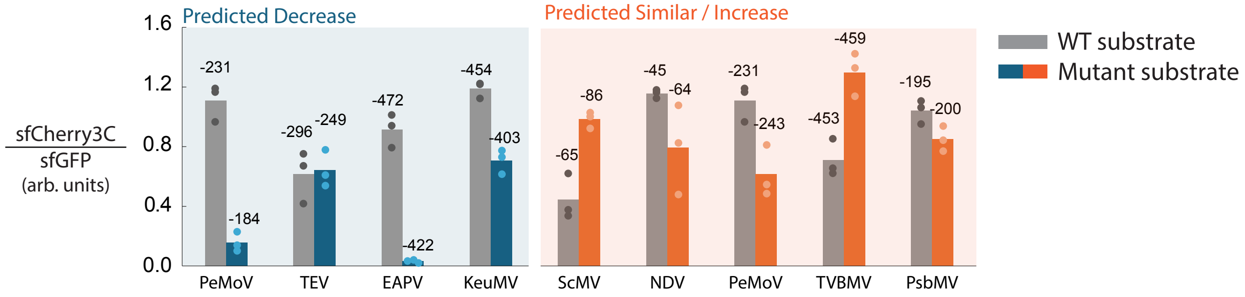

Supplementary Figure 5: Predicted and experimentally measured proteolytic cleavage of cognate and point-mutant substrates. H_{spec} scores are shown above the bars. Proteases tested against mutant substrates are predicted to exhibit a decrease (blue) or increase (orange) in cleavage-induced fluorescence when compared to that of wild-type (WT) substrate (grey). For each protease, mutants contained the following mutations (left to right): PeMoV cleavage site (cs) Y(P2)H; TEVcs L(P4)V; EAPVcs L(P2)H; KeuMVcs L(P2)H; ScMVcs F(P3)Y; NDVcs S(P1')A; PeMoVcs S(P1')V; TVBMVcs N(P1')G; PSbMVcs V(P4). Bars represent the means of 3 independent experiments. Arb. units, arbitrary units. Source data are provided as a Source Data file.

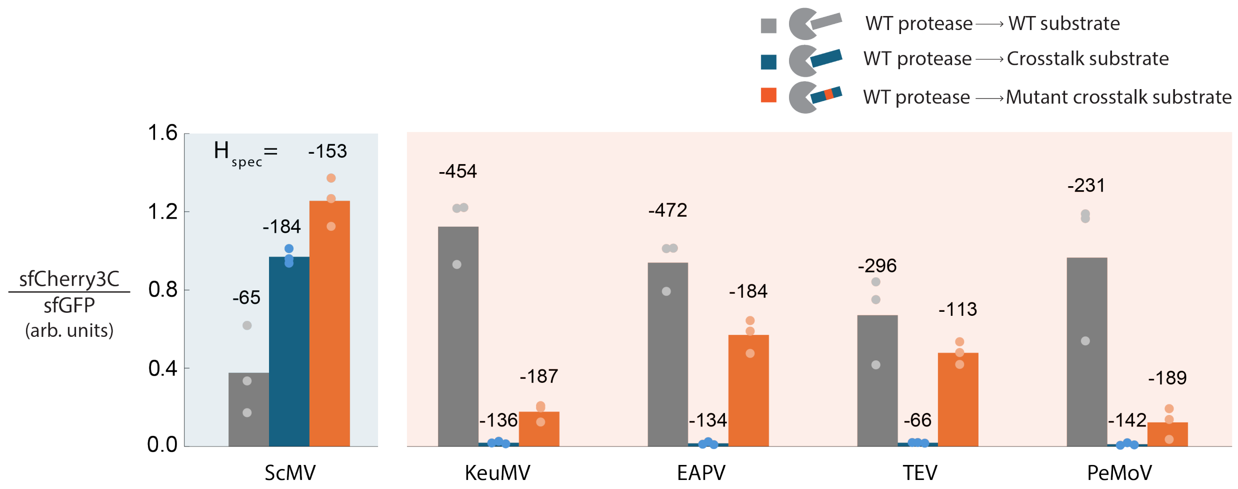

Supplementary Figure 6: ProSSpeC can prescribe single-site mutations that elicit crosstalk between protease and substrate that do not originally interact. ProSSpeC H_{spec} scores above bars predict the favorable interaction of each protease-substrate pair. For example, left plot shows that crosstalk interaction between ScMVp with NLSYVcs (blue) was predicted to be stronger than when ScMVp is paired with mutant NLSYVcs (orange). Right plot: proteases tested against all other crosstalk substrates (blue) were predicted to exhibit increased cleavage-induced fluorescence when the crosstalk substrates are mutated (orange). The tested proteases and substrates are as follows (left to right): ScMVp was tested against wild-type (WT) ScMVcs, NLSYVcs, and mutant NLSYVcs E(P5)T. KeuMVp was tested against WT KeuMVcs, PPVcs, and mutant PPVcs H(P2)L. EAPVp was tested against WT EAPVcs, SPV2cs, and mutant SPV2cs H(P2)L. TEVp was tested against WT TEVcs, TVMVcs, and mutant TVMVcs V(P4)L. PeMoVp was tested against WT PeMoVcs, LMVcs, and mutant LMVcs H(P2)Y (see Table S5 for more details). Bars represent the means of 3 independent experiments. Arb. units, arbitrary units. Source data are provided as a Source Data file.

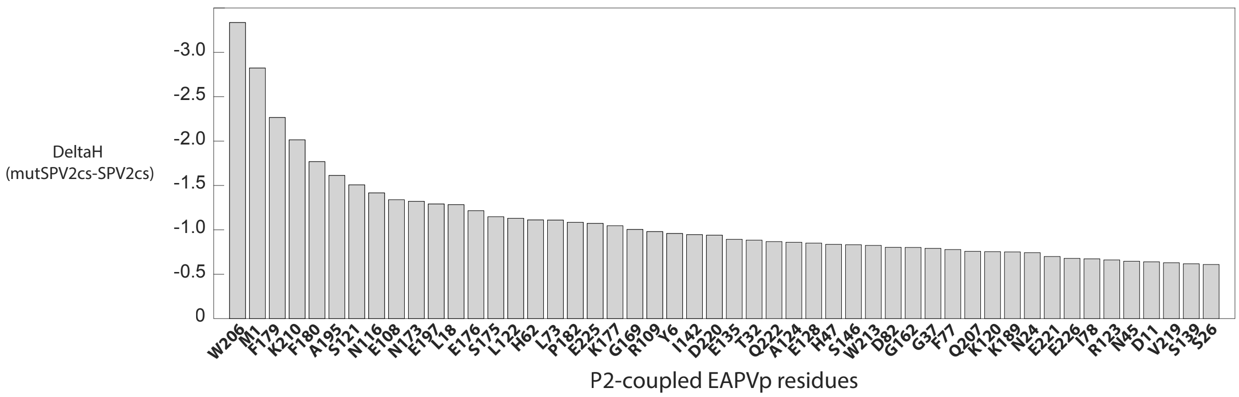

Supplementary Figure 7: Top 50 most negative DeltaH of P2-coupled EAPVp residues between EAPVp-SPV2cs and EAPVp-mutSPV2cs. Aligned residue pairs between EAPVp and P2 (i-j) are arranged left to right in decreasing contribution to H_{spec} score difference between the two protease-substrate pairs. Source data are provided as a Source Data file.

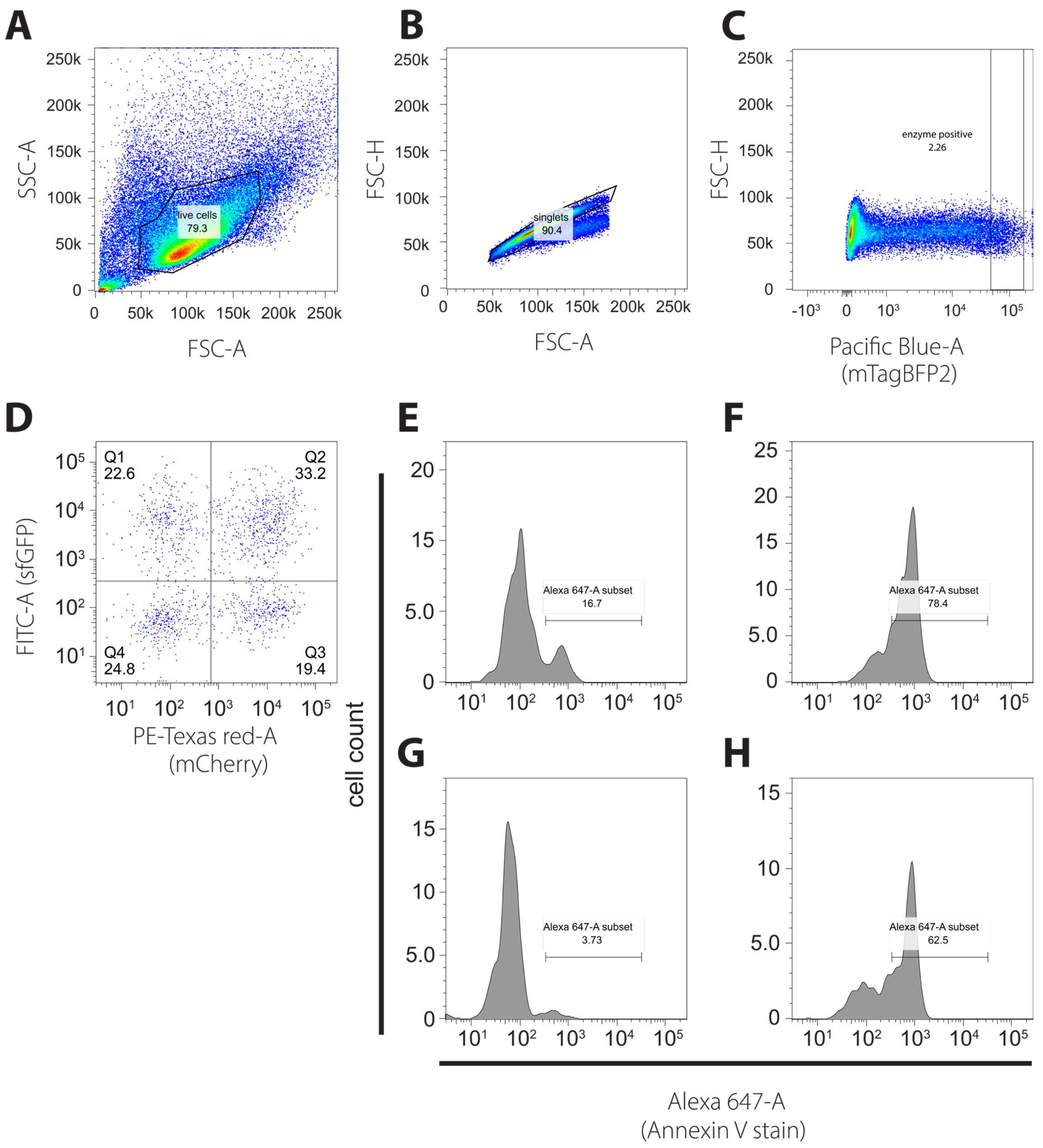

Supplementary Figure 8: Gating strategy for selective synpoptosis of mutant cells in a mixed cell population. Cells were co-transfected (see Methods) with sfGFP-tagged Caspase 3 containing the SPV2cs: AVESNDCEPVY**H**QSGTEETK, mCherry-tagged Caspase 3 containing the mutated SPV2cs: AVESNDCEPVY**L**QSGTEETK, and mTagBFP2-tagged EAPVp. Transfected cells were analyzed via flow cytometry and gated for (**A**) cells, (**B**) then singlets, (**C**) then EAPVp-positive (mTagBFP2+) cells. (**D**) Cell types were then separately analyzed: cells without any Caspase 3 (Q4), with only sfGFP-Caspase3-SPV2cs (Q1), with only mCherry-Caspase3-mutSPV2cs (Q3), or with both Caspase3 constructs (Q2). **E**-**H** are Q1-Q4 histograms of cells stained with Alexa 647-conjugated Annexin V, which labels apoptotic cells. A similar gating strategy was done for cells transfected with SPV2p instead of EAPVp except on **(C),** where middle expression of SPV2 was chosen.

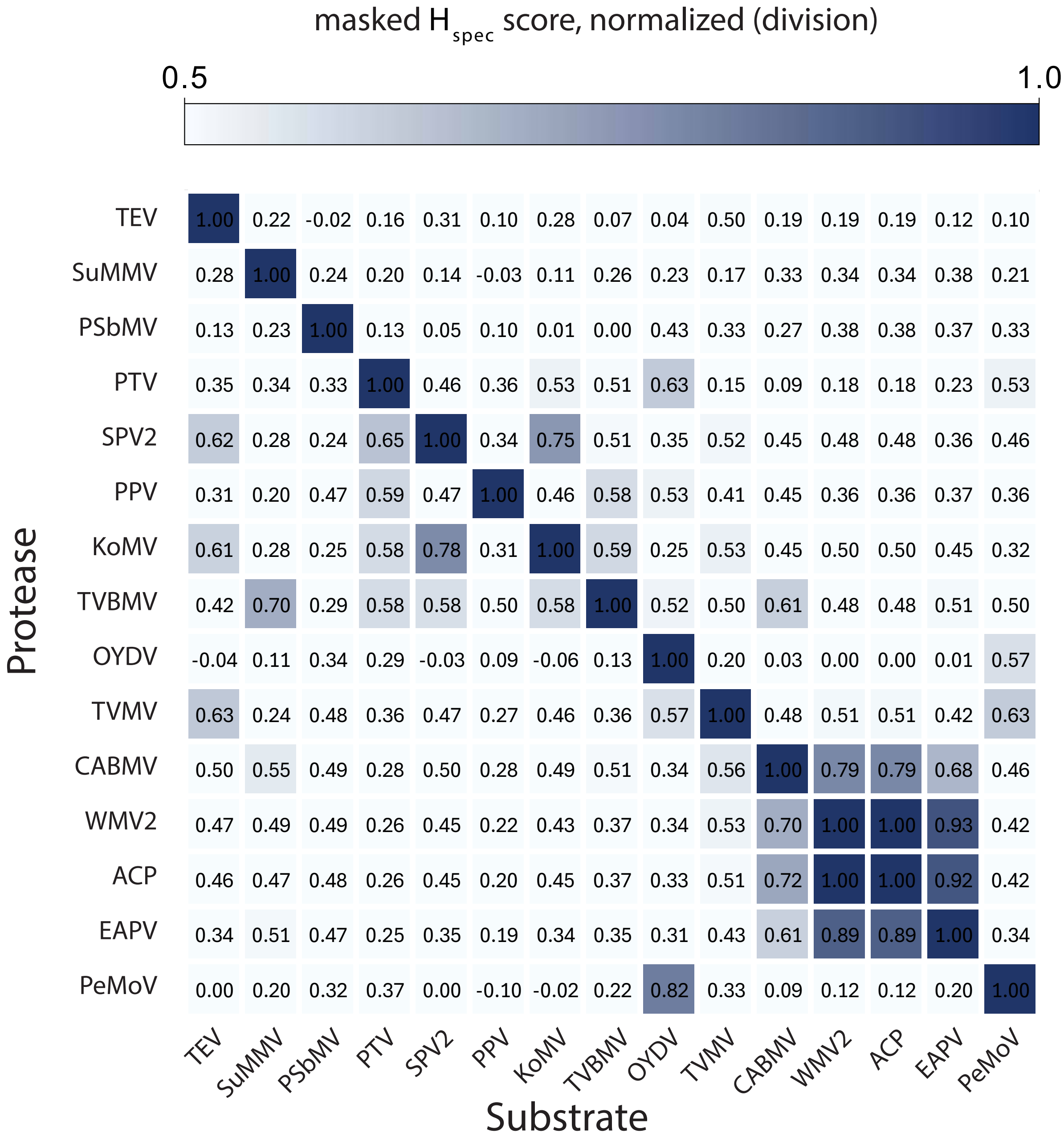

Supplementary Figure 9: Orthogonality matrix of normalized masked H_{spec} scores. Masked H_{spec} scores were normalized relative to each protease's cognate substrate masked H_{spec} score. Scores were normalized by division (Equation 5), representing each interaction as a fraction of the cognate cleavage strength. Smaller ratios indicate weaker cross-reactivity. Diagonal values correspond to protease–substrate cognate-pairs used as normalization references. Source data are provided as a Source Data file.

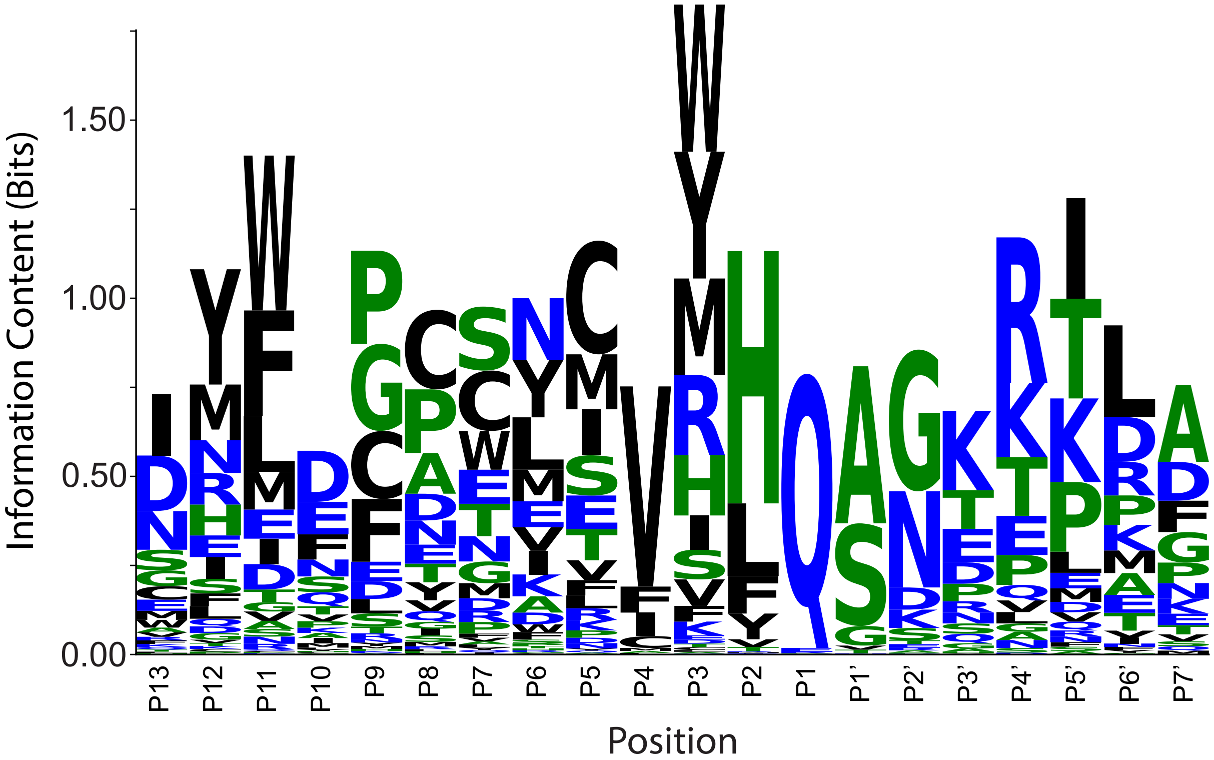

Supplementary Figure 10: Sequence logo of NIa-protease substrates used for concatenated alignment. The logo shows that glutamine (Q) is significantly conserved at P1. Sequences were aligned to their corresponding hmm profile and then concatenated if they belonged to the same polyprotein. Source data are provided as a Source Data file.

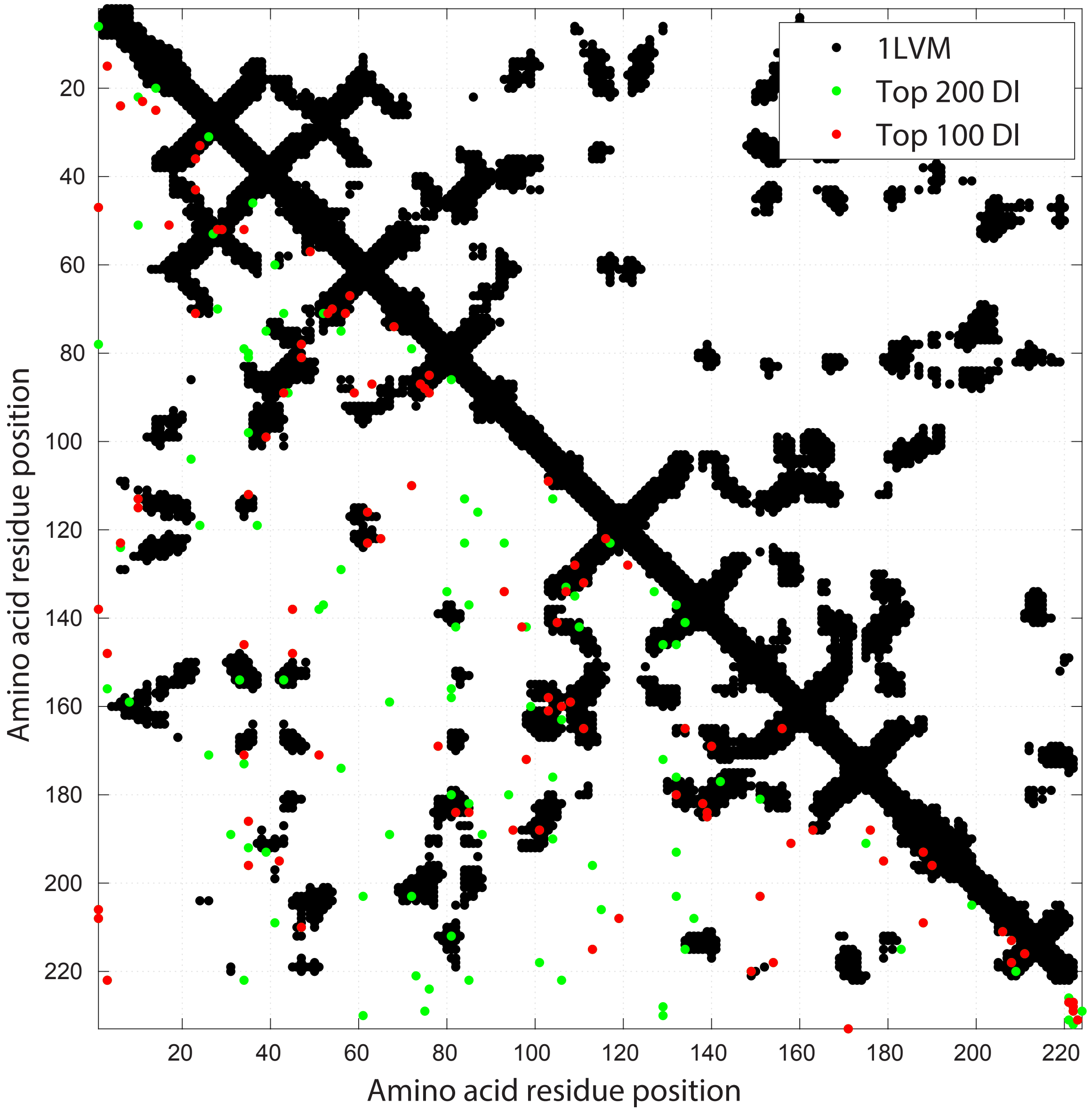

Supplementary Figure 11: Top direct information (DI) contacts in the monomeric structure of TEVp. Many structural contacts (distance ≤ 10 Å, black) of TEVp (PDB ID: 1LVM) are captured by DCA (red: Top 100 DI, and green: Top 200 DI). Source data are provided as a Source Data file.

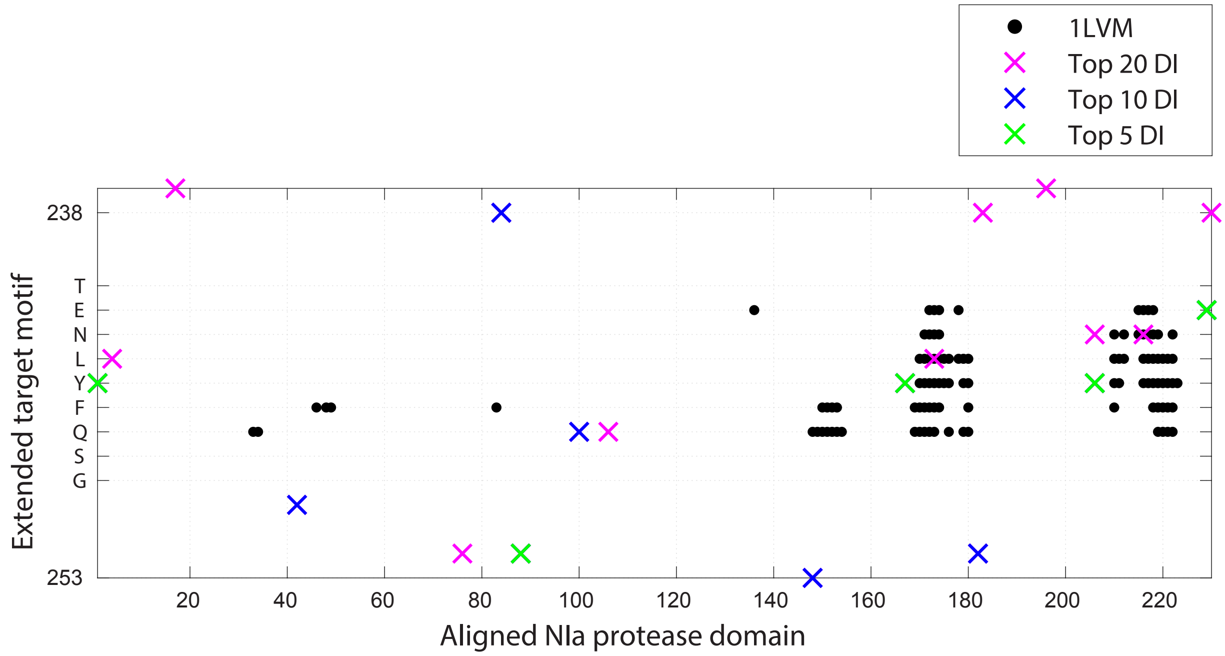

Supplementary Figure 12: Interfacial DI contacts between TEVp and its 7-amino-acid substrate. These structural contacts (black dots) at the interface of TEVp and its substrate (PDB ID: 1LVM) show overlap with the top DI pairs captured by DCA (crosses). Note that the substrate structure in 1LVM is only 9 residues long (TENLYFQSG), so DI pairs outside of this range could not be validated. 1LVM represents only one conformation of the protease-substrate complex, so it is possible that DI pairs in the 7-amino acid substrate range that are not validated by 1LVM are still important. Source data are provided as a Source Data file.

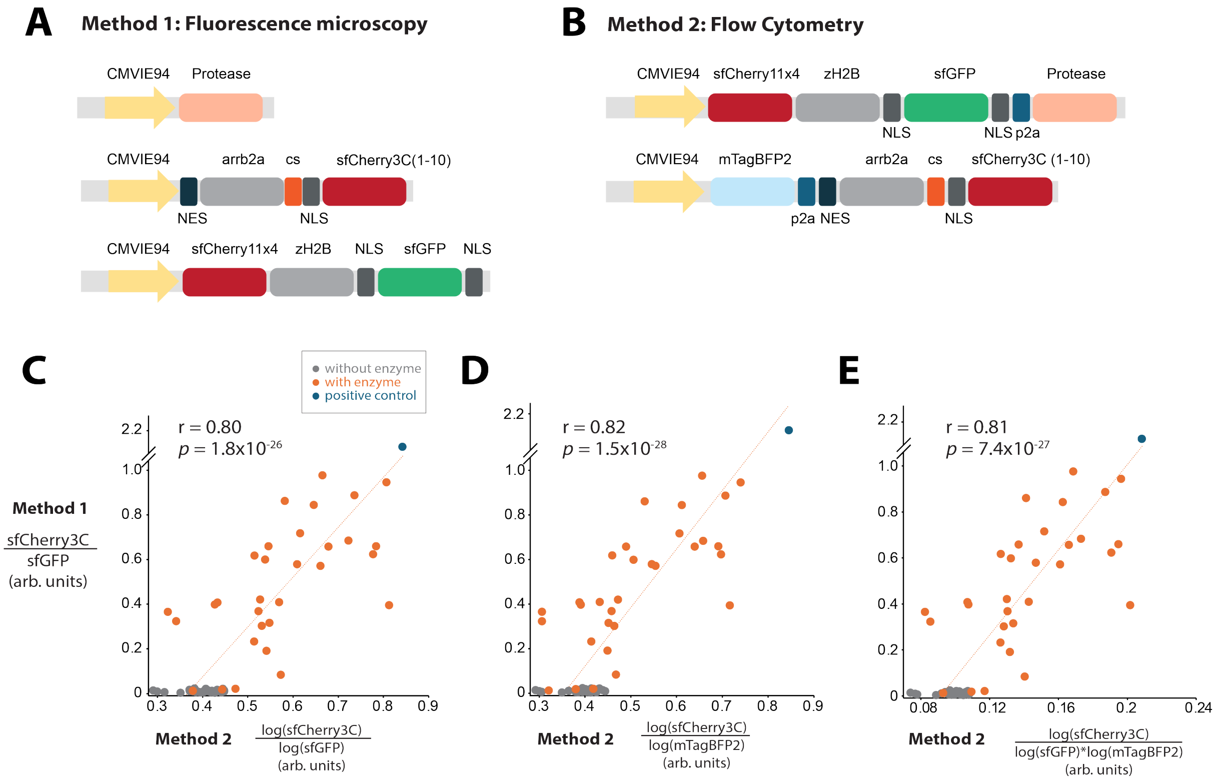

Supplementary Figure 13: Comparison between fluorescence microscopy and flow cytometry for quantifying cleavage-induced fluorescence. (**A**) Constructs used for fluorescence microscopy. Only the nuclear reporter has a fluorescent protein tag (sfGFP). (**B**) Constructs used for flow cytometry. Protease was fused to the nuclear reporter while the substrate was tagged with mTagBFP2. **(C-E)** Comparison of method 1 (y-axis) vs method 2 (x-axis) with cleavage-induced fluorescence normalized against protease amount (**C**), substrate amount (**D**), or both (**E**). In all cases, method 1 and method 2 show strong positive correlations (Pearson r = 0.80, 0.82, 0.81; one-tailed p = 1.8×10^{-26}, 1.5×10^{-28}, 7.4×10^{-27}, respectively). Each dot represents the mean of 3 independent experiments. Arb. units, arbitrary units. Source data are provided as a Source Data file.

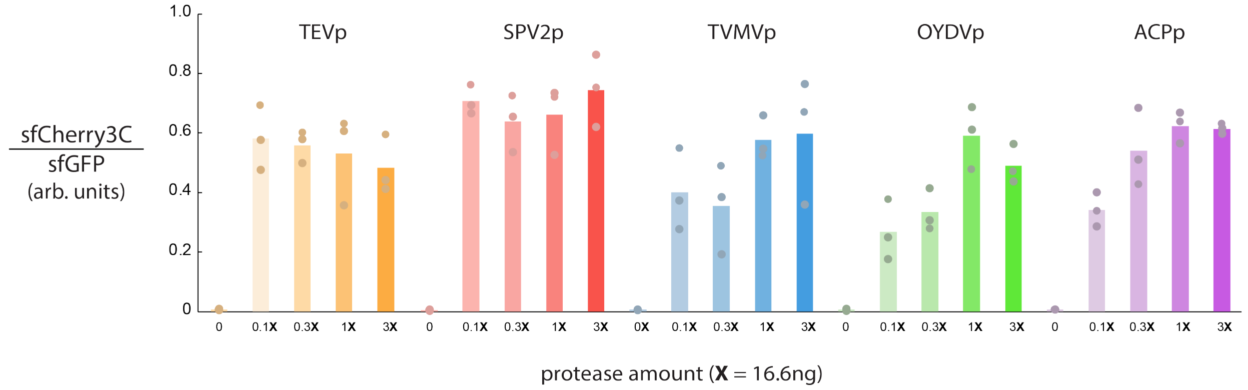

Supplementary Figure 14: Protease titration. Bars represent cleavage-induced fluorescence as a function of protease amount. For each protease, increasing amounts are indicated by increasing color saturation. Bars represent the means of 3 independent experiments. Arb. units, arbitrary units. Source data are provided as a Source Data file.

Supplementary Table 1: Equivalent positions of TEVp(S219V). Most serines were mutated to valine.

Protease	Equivalent Position	Tested Amino Acid	Wild Type Amino Acid
SPV2	221	V	S
TVMV	219	D	D
OYDV	221	D	D
ACP	222	E	E
PeMoV	221	N	N
PTV	221	V	S
SuMMVp	219	D	D
WMV2	222	E	E
SPFMV	221	G	G
PPV	221	D	D
PS	220	V	S
NDV	221	D	D
TVBMV	220	V	S
KeuMV2	222	D	D
TEV	219	V	S
CABMV	221	N	N
SYSVp	219	G	G
ClYVV	221	D	D
AWMV	219	V	S
WTMV	220	D	D
ScMV	221	V	S
EAPV	221	E	E
KoMVp	219	K	K
PSbMV	222	N	N
KeuMV	220	D	D
LMoVp	219	D	D
ZYMV	221	E	E
LMV	221	V	S
PMMV	222	D	D
ISMV	222	S	S
PMAV	220	V	S

Supplementary Table 2: Masked versus Full H_{spec} for all natural proteases tested against their cognate 7-residue substrates. Masked H_{spec} scores pertain to the interaction between a protease and its 7-residue substrate, whereas Full H_{spec} scores pertain to the interaction between a protease and the extended 20-residue substrate. In many cases, but not all, extending the substrate context surrounding P6 - P1′ improves H_{spec}. Normalized fluorescence values are from three independent experiments.

Enzyme	Normalized fluorescence (sfCherry3C/sfGFP)	Masked H_{spec}	Full H_{spec}	DeltaH (Full - Masked)
TVMVp	**0.97705074**	-172.20961	-492.78064	-320.57103
SPV2p	**0.94568743**	-135.28855	-402.90842	-267.61987
PeMoVp	**0.88775065**	-124.63403	-231.20805	-106.57402
OYDVp	**0.86160768**	-148.04855	-400.57976	-252.5312
ACPp	**0.84422417**	-211.24247	-409.10627	-197.8638
PTVp	**0.71701207**	-91.580532	-332.16211	-240.58157
SuMMVp	**0.68467667**	-186.63381	-499.71946	-313.08565
SPFMVp	**0.66015189**	-121.54826	-359.4278	-237.87954
PPVp	**0.65890292**	-154.56169	-358.24135	-203.67966
WMV2p	**0.65765055**	-212.61772	-357.44387	-144.82615
TEVp	**0.62399523**	-147.88353	-296.63151	-148.74798
KeuMV2p	**0.61852635**	-212.61772	-455.38367	-242.76595
NDVp	**0.59955501**	-50.480701	-45.043885	5.43681624
TVBMVp	**0.57978388**	-153.61923	-453.87838	-300.25915
PSp	**0.5721828**	-213.69393	-501.15486	-287.46093
WTMVp	**0.42105511**	-156.51053	-449.03081	-292.52028
CABMVp	**0.40963172**	-179.48055	-402.98447	-223.50391
SYSVp	**0.40778096**	-118.17245	-412.23046	-294.05801
AWMVp	**0.39816353**	-135.11726	-442.112	-306.99474
KoMVp	**0.39517893**	-134.31441	-442.06288	-307.74847
ScMVp	**0.36854913**	-58.899314	-65.414463	-6.5151495
ClYVVp	**0.36589698**	-188.01749	-472.72616	-284.70866
PSbMVp	**0.32388596**	-145.7161	-195.96427	-50.248171
KeuMVp	**0.31634571**	-196.10263	-454.69011	-258.58748
EAPVp	**0.30268607**	-198.68661	-472.55665	-273.87004
LMoVp	**0.23324098**	-187.64189	-457.21893	-269.57705
ZYMVp	**0.19182977**	-172.68205	-412.35792	-239.67588
LMVp	**0.08469378**	-138.0905	-364.20233	-226.11183
PMMVp	**0.02116709**	-217.23906	-546.39607	-329.15701
PMAVp	**0.01834021**	-213.09107	-483.22047	-270.12941
ISMVp	**0.01220867**	-214.61812	-502.02259	-287.40447

Supplementary Table 3: Predicted ΔH_{spec} (DeltaH) and corresponding change in fluorescence (DeltaFluor) between the sample pair and the reference pair. DeltaFluor mean and standard error (S.E.) values are from three independent experiments.

Sample pair	Reference pair	DeltaH	DeltaFluor	S.E.
**ScMVp-mutScMVcs F(P3)Y**	ScMVp-ScMVcs	-21.27	0.55	0.16
**NDVp-mutNDVcs S(P1')A**	NDVp-NDVcs	-19.36	-0.34	0.18
**PeMoVp-mutPeMoVcs S(P1')V**	PeMoVp-PeMoVcs	-12.50	-0.42	0.25
**TVBMVp-mutTVBMVcs N(P1')G**	TVBMVp-TVBMVcs	-6.00	0.67	0.14
**PSbMVp-mutPSbMVcs V(P4)A**	PSbMVp-PSbMVcs	-4.28	-0.11	0.14
**KeuMVp-mutKeuMVcs L(P2)H**	KeuMVp-KeuMVcs	50.79	-0.51	0.17
**EAPVp-mutEAPVcs L(P2)H**	EAPVp-EAPVcs	49.82	-0.91	0.07
**TEVp-mutTEVcs L(P4)V**	TEVp-TEVcs	47.39	-0.06	0.15
**PeMoVp-mutPeMoVcs Y(P2)H**	PeMoVp-PeMoVcs	46.94	-0.83	0.22
**KeuMVp-mutPPVcs H(P2)L**	KeuMVp-PPVcs	-50.79	0.16	0.03
**EAPVp-mutSPV2cs H(P2)L**	EAPVp-SPV2cs	-49.82	0.55	0.05
**TEVp-mutTVMVcs V(P4)L**	TEVp-TVMVcs	-47.39	0.46	0.03
**PeMoVp-mutLMVcs H(P2)Y**	PeMoVp-LMVcs	-46.94	0.11	0.05
**ScMVp-mutNLSYVcs E(P5)T**	ScMVp-NLSYVcs	30.72	0.29	0.07
**KeuMVp-mutPPVcs H(P2)L**	KeuMVp-KeuMVcs	267.49	-0.95	0.10
**EAPVp-mutSPV2cs H(P2)L**	EAPVp-EAPVcs	287.74	-0.37	0.09
**TEVp-mutTVMVcs V(P4)L**	TEVp-TEVcs	182.98	-0.19	0.13
**PeMoVp-mutLMVcs H(P2)Y**	PeMoVp-PeMoVcs	41.40	-0.84	0.22
**ScMVp-mutNLSYVcs E(P5)T**	ScMVp-ScMVcs	-88.57	0.88	0.15
**KeuMVp-PPVcs**	KeuMVp-KeuMVcs	318.28	-0.95	0.10
**EAPVp-SPV2cs**	EAPVp-EAPVcs	337.56	-0.92	0.07
**TEVp-TVMVcs**	TEVp-TEVcs	230.38	-0.65	0.13
**PeMoVp-LMVcs**	PeMoVp-PeMoVcs	88.34	-0.95	0.21
**ScMVp-NLSYVcs**	ScMVp-ScMVcs	-119.29	0.59	0.13

Supplementary Table 4: *Potyviridae* sequences utilized in this study. Viruses with an asterisk were used to build the *hmm* family for the substrate sequences and align the remaining substrate sequences. The seed alignment was created by aligning known substrates at the P1 position and capturing a 20-amino-acid window.

Abbreviation	Name	Uniprot ID	Substrate
**ACP**	Artemisia carvifolia potyvirus	A0A7D3QY18	NHTDGCCESVSLQSGKEKET
**AMoV***	Arracacha mottle virus	A1C2E6	VFHENQHSLVYHQGDNQTVD
**AspV1**	Asparagus virus 1	A0A0A7DZP8	DEESEAIFEVSHQANDTVDA
**AWMV***	Algerian watermelon mosaic virus	B2Y6H0	EAGDTPELVVYHQADTPQDA
**BaRMV***	Basella rugose mosaic virus	A6XJ24	INDNEQEDEVVYQSGSGPAT
**BBMV**	Banana bract mosaic virus	A6XMW6	GRKFITDTDEIFQSLNMKAA
**BCMV***	Bean common mosaic virus	Q7THD2	DYEVGCGESVHLQSGIGQPQ
**BsMoV***	Brugmansia suaveolens mottle virus	E1CHK3	EDFECGCYEVRHQSTTSTPA
**CABMV***	Cowpea aphid-borne mosaic virus	F5BFU0	FYDDCESEDVVLQSDEKQKE
**CDV***	Colombian datura virus	L0H5K6	YDDSLASEDIYLQADTVDAG
**ChiRSV***	Chilli ringspot virus	G5CBY6	SQWKDEDTIVYHQADTQAVD
**ClYVV**	Clover yellow vein virus	A0A7H5KCL7	QHDEFDDMKFVFQSDKEKLN
**EAPV***	East Asian Passiflora distortion virus	Q2MH09	EHVEGCCESVSLQTKSEENK
**ERV**	Euphorbia ringspot virus	A0A1D8FFY0	QEIIMCKAKLAFQKLQTPVL
**HbPV1**	Hubei Poty-like virus 1	A0A1L3KNS8	EPESDEVLVCRFQANQERLN
**ISMV**	Iris severe mosaic virus	A0A2U8JDD4	LHDTNPIINVRFEGDSEDAA
**KeuMV* / KeuMV2**	Keunjorong mosaic virus	G8EG34	DRDIGEEDVVTLQSGEEKDA
**KoMV**	Konjac mosaic virus	Q25C85	NIEWPQEEQVYHQSGKEEKD
**LMoV**	Lily mottle virus	D7UTC2	EGECGGAEIVAFQANETLNT
**LMV***	Lettuce mosaic virus	P89876	DEDDDDMDEVYHQVDTKLDA
**LEPV**	Lycopersicon esculentum potyvirus	A0A6M8PU83	DEFECDTYEVHHQANDTIDA
**LYSV***	Leek yellow stripe virus	M9PQ66	DSDAPIEETFVFQANDELDA
**MWMV***	Moroccan watermelon mosaic virus	A8S2H4	ESGDCPELMVYHQADDARDA
**NDV**	Narcissus degeneration virus	I2E5K2	LNEPDIXQRVSFQSGEELDA
**NLSYV***	Narcissus late season yellows virus	I3VPR2	DFDDKTPLEVYHQSGKQTLD
**OYDV**	Onion yellow dwarf virus	G9FIG6	ENEEVVPKEVRYQAGKGEDA
**PeMoV**	Peanut mottle virus	O56075	DEDEDHNDEVRYQSGENKSK
**PenMV***	Pennisetum mosaic virus	Q4VS22	GYVEDYNEDVYHQSGRGDAG
**PepSMV***	Pepper severe mottle virus	Q0E7B2	DELECRPYEVYHQADTTVDA
**PkMV***	Pokeweed mosaic virus	J3SYI6	DELTDEQIEVYFQSDVNAGV
**PMAV**	Pecan mosaic-associated virus	A0A173GTP8	RYMHDEFDSITFQANETDET
**PMMV**	Platycodon mild mottle virus	A0A385MJH8	LGLQDLAEPMKFQVDETLDA

**PPV***	Plum pox potyvirus	P13529	INDDGESNVVVHQADEREDE
**PS**	Potyvirus sp	A0A6B9KS84	EDSEQEIEIVDFQADTKSTG
**PSbMV**	Pea seed-borne mosaic virus	P29152	DEGGDGSIKVRLQAGDETKD
**PTV***	Peru tomato mosaic virus	Q80P26	DELECDSDEVYHQAKDDSGT
**PVB***	Potato virus B	A0A2U5BZQ5	EDETEDEDTVFFQAGTLDAG
**PVY***	Potato virus Y	P18247	DEFELDSYEVHHQANDTIDA
**ScMV***	Sugarcane mosaic virus	Q8QQV9	GYIEDYNEDVFHQSGTVDAG
**SPFMV***	Sweet potato feathery mottle virus	D4Q9P1	SMLQENELEVYHQSGEKTEF
**SPV2***	Sweet potato virus 2	I2CMD1	AVESNDCEPVYHQSGTEETK
**SPVG***	Sweet potato virus G	I6QL83	SLTNFEDNNVHHQSAEEIYD
**SrMV***	Sorghum mosaic virus	P89208	EYIEDELIDVRHQAGGGTVD
**SuCMoV***	Sunflower chlorotic mottle virus	D5J730	DEFECGTYEVHHQGDNIDAG
**SuMMV**	Sunflower mild mosaic virus	M9NYB7	IDDGMKYEEIHLQGDKVNAG
**SYSV**	Shallot yellow stripe virus	Q3LE82	DDRIPTPSYVSYQASESEDA
**TEV**	Tobacco etch virus	P04517	DYDIPTTENLYFQSGTVDAG
**TuMV2**	Turnip mosaic virus	Q7T469	DYEDGTEACVYHQAGETLDA
**TVBMV***	Tobacco vein banding mosaic virus	A8QWY6	NQWKDEQEEVVHQNDEQTVD
**TVMV**	Tobacco vein mottling virus	P09814	ANNEFLRETVRFQSDTVDAG
**WMV**	Watermelon mosaic virus	A0A6G8QIN0	NHTDGCCESVSLQSGKEKET
**WMV2**	Watermelon mosaic virus	A0A0A7CBE0	SHIDSCCESVSLQSGKEAVE
**WPMV***	Wild potato mosaic virus	Q8B6S3	CDSFEVRHRANEEGGTLDTG
**WTMV***	Wild tomato mosaic virus	A6XMW5	NEHSSDDLDVRHQSGETVDA
**ZYMV***	Zucchini yellow mosaic virus	Q2MHN6	DTFFEQGDTVMLQSGTQPTV

Supplementary Table 5: Gated flow cytometry cell counts capture protease-induced, caspase-mediated apoptosis (synpoptosis). Cells were stained with Alexa Fluor 350-conjugated Annexin V, which labels apoptotic cells (DAPI channel). Cells were co-transfected with sfGFP-Caspase 3-SPV2cs (FITC channel) or with mCherry-Caspase 3-mutSPV2cs (PE-Texas Red channel), and with or without EAPVp or SPV2p. For each gate, cell count percentages are calculated from the parent population (live, singlet cells). The fold-differences between % Annexin V+ cells with or without protease (that can cleave Caspase 3) are highest in conditions with 20 ng Caspase 3 (Q2, underlined). A total of 20,000 cells were analyzed per condition.

Protease	Substrate (cleavage sequence in caspase 3)	Caspase 3 amount (ng)	Q1 High DAPI, low (FITC or PE-Texas red)	Q2 High DAPI, High (FITC or PE-Texas red)	Q3 Low DAPI, Low (FITC or PE-Texas red)	Q4 Low DAPI, High (FITC or PE-Texas red)
None	mutSPV2cs	5	1.10%	0.80%	66.90%	31.30%
None	mutSPV2cs	20	0.70%	<u>1.10%</u>	46%	52.20%
None	mutSPV2cs	100	0.90%	4%	34%	61.20%
EAPVp	mutSPV2cs	5	5.80%	8.20%	68.50%	17.40%
EAPVp	mutSPV2cs	20	6%	<u>14.20%</u>	48.40%	31.40%
EAPVp	mutSPV2cs	100	5.20%	19.50%	28.30%	47%
SPV2p	mutSPV2cs	5	2.90%	1.60%	83.20%	12.30%
SPV2p	mutSPV2cs	20	1.70%	<u>6.50%</u>	55.70%	36.10%
SPV2p	mutSPV2cs	100	1.80%	14.90%	33.10%	50.10%
None	SPV2cs	5	10.70%	5.10%	51.10%	33.10%
None	SPV2cs	20	3.60%	<u>9.70%</u>	27.50%	59.20%
None	SPV2cs	100	1.60%	17.40%	11.20%	69.80%
EAPVp	SPV2cs	5	5.20%	3.90%	58.10%	32.80%
EAPVp	SPV2cs	20	3.80%	<u>11.20%</u>	34.10%	50.90%
EAPVp	SPV2cs	100	1.50%	18.10%	15.50%	64.90%
SPV2p	SPV2cs	5	6.10%	3.60%	63.50%	26.80%
SPV2p	SPV2cs	20	11.40%	<u>21.10%</u>	53.50%	14.10%
SPV2p	SPV2cs	100	9.20%	30.40%	48.10%	12.30%
