# Supplementary material for: Identification and engineering of highly functional potyviral proteases in cells using co-evolutionary models
Source: Nat Commun. 2026 Feb 26;17:3257. doi: 10.1038/s41467-026-69961-5 (PMC13062108; doi:10.1038/s41467-026-69961-5)
Supplement: Supplementary file 3 — Description of Additional Supplementary Files [file 41467_2026_69961_MOESM3_ESM.pdf]

Description of Additional Supplementary Files

Supplementary Data 1: Overview of aligned sequences from Potyviridae.

Supplementary Data 2: List of plasmids used in this study.
