# Supplementary material for: Identification and engineering of highly functional potyviral proteases in cells using co-evolutionary models
Source: Nat Commun. 2026 Feb 26;17:3257. doi: 10.1038/s41467-026-69961-5 (PMC13062108; doi:10.1038/s41467-026-69961-5)
Supplement: Supplementary file 6 — Reporting Summary [file 41467_2026_69961_MOESM6_ESM.pdf]

Corresponding author(s): P. C. Dave P. Dingal, Faruck Morcos

Last updated by author(s): Nov 11, 2025

Reporting Summary

Nature Portfolio wishes to improve the reproducibility of the work that we publish. This form provides structure for consistency and transparency in reporting. For further information on Nature Portfolio policies, see our Editorial Policies and the Editorial Policy Checklist.

Statistics

For all statistical analyses, confirm that the following items are present in the figure legend, table legend, main text, or Methods section.

n/a	Confirmed	
☐	☒	The exact sample size (*n*) for each experimental group/condition, given as a discrete number and unit of measurement
☐	☒	A statement on whether measurements were taken from distinct samples or whether the same sample was measured repeatedly
☐	☒	The statistical test(s) used AND whether they are one- or two-sided<br>*Only common tests should be described solely by name; describe more complex techniques in the Methods section.*
☐	☒	A description of all covariates tested
☒	☐	A description of any assumptions or corrections, such as tests of normality and adjustment for multiple comparisons
☐	☒	A full description of the statistical parameters including central tendency (e.g. means) or other basic estimates (e.g. regression coefficient) AND variation (e.g. standard deviation) or associated estimates of uncertainty (e.g. confidence intervals)
☐	☒	For null hypothesis testing, the test statistic (e.g. *F*, *t*, *r*) with confidence intervals, effect sizes, degrees of freedom and *P* value noted<br>*Give P values as exact values whenever suitable.*
☒	☐	For Bayesian analysis, information on the choice of priors and Markov chain Monte Carlo settings
☒	☐	For hierarchical and complex designs, identification of the appropriate level for tests and full reporting of outcomes
☐	☒	Estimates of effect sizes (e.g. Cohen's *d*, Pearson's *r*), indicating how they were calculated

Our web collection on statistics for biologists contains articles on many of the points above.

Software and code

Policy information about availability of computer code

Data collection	Protease and substrate sequences were gathered using Matlab 2023b and hmmer 3.4.
Data analysis	Hspec scores were calculated using code posted in this link: https://github.com/morcoslab/ProSSpeC/. In silico plots were generated usingPython 3.11.5 (scipy 1.11.1, matplotlib 3.7.2, biopython 1.78, pandas 2.1.1, numpy 1.24.3, ete3 3.1.3). Fluorescence microscopy images were analyzed with ImageJ software (v 2.14.0/1.54f). For all flow cytometry experiments, flow cytometry data were processed using FlowJo software (v10.10.0).

For manuscripts utilizing custom algorithms or software that are central to the research but not yet described in published literature, software must be made available to editors and reviewers. We strongly encourage code deposition in a community repository (e.g. GitHub). See the Nature Portfolio guidelines for submitting code & software for further information.

Data

Policy information about availability of data

All manuscripts must include a data availability statement. This statement should provide the following information, where applicable:

- Accession codes, unique identifiers, or web links for publicly available datasets
- A description of any restrictions on data availability
- For clinical datasets or third party data, please ensure that the statement adheres to our policy

The data generated in this study have been deposited in the Zenodo database under accession code https://doi.org/10.5281/zenodo.15039890. The overview of

aligned Potyviridae sequences and the list of plasmids (Supplementary data 2) used in this study are provided as Supplementary Data. The plasmid sequences and maps for all proteases, the 7-, GS-flanked 7-, and 20-amino acid substrate of TEVp, and the H2B-sfGFP reporter used here are available on Addgene: https://www.addgene.org/Dave_Dingal/. Source data are provided with this paper. The protein structural data used in this study are available in the PDB database under accession code 1LVM [https://www.rcsb.org/structure/1LVM]. The protein family profile HMM used in this study are available in the InterPro database under accession code PF00863 [https://www.ebi.ac.uk/interpro/entry/pfam/PF00863/logo/].

Research involving human participants, their data, or biological material

Policy information about studies with human participants or human data. See also policy information about sex, gender (identity/presentation), and sexual orientation and race, ethnicity and racism.

Reporting on sex and gender	N/A
Reporting on race, ethnicity, or other socially relevant groupings	N/A
Population characteristics	N/A
Recruitment	N/A
Ethics oversight	N/A

Note that full information on the approval of the study protocol must also be provided in the manuscript.

Field-specific reporting

Please select the one below that is the best fit for your research. If you are not sure, read the appropriate sections before making your selection.

☒ Life sciences ☐ Behavioural & social sciences ☐ Ecological, evolutionary & environmental sciences

For a reference copy of the document with all sections, see nature.com/documents/nr-reporting-summary-flat.pdf

Life sciences study design

All studies must disclose on these points even when the disclosure is negative.

Sample size	All cell culture experiments were performed with 2 technical replicates through transfection of HEK293 cells and 30 cells for each replicate were analyzed.
Data exclusions	No exclusion was done in all experiments.
Replication	Each experiment was repeated at least three times The number of replicates done for each specific experiment is detailed in the figure captions.
Randomization	Images were taken from a random position in the wells for all cell culture experiments.
Blinding	No, the experimenter designed and conducted the experiments themselves but the analyzed cells were selected at random.

Reporting for specific materials, systems and methods

We require information from authors about some types of materials, experimental systems and methods used in many studies. Here, indicate whether each material, system or method listed is relevant to your study. If you are not sure if a list item applies to your research, read the appropriate section before selecting a response.

Materials & experimental systems

n/a	Involved in the study	
☒	☐	Antibodies
☐	☒	Eukaryotic cell lines
☒	☐	Palaeontology and archaeology
☒	☐	Animals and other organisms
☒	☐	Clinical data
☒	☐	Dual use research of concern
☒	☐	Plants

Methods

n/a	Involved in the study	
☒	☐	ChIP-seq
☐	☒	Flow cytometry
☒	☐	MRI-based neuroimaging

Eukaryotic cell lines

Policy information about cell lines and Sex and Gender in Research

Cell line source(s)	We used HEK293 cell line (ATCC, RRID:CVCL_0045).
Authentication	The cell line was not additionally authenticated.
Mycoplasma contamination	The cell line was not tested for mycoplasma contamination.
Commonly misidentified lines (See ICLAC register)	No commonly misidentified cell lines were used.

Plants

Seed stocks	N/A
Novel plant genotypes	N/A
Authentication	N/A

Flow Cytometry

Plots

Confirm that:

☒ The axis labels state the marker and fluorochrome used (e.g. CD4-FITC).

☒ The axis scales are clearly visible. Include numbers along axes only for bottom left plot of group (a 'group' is an analysis of identical markers).

☒ All plots are contour plots with outliers or pseudocolor plots.

☒ A numerical value for number of cells or percentage (with statistics) is provided.

Methodology

Sample preparation	The cells were stained in 1X Annexin Binding buffer (ThermoFisher Scientific, cat. no. V13246). No wash step was done before analysis.
Instrument	Flow cytometry analysis was done using BD LSRFortessa™ Cell Analyzer. The following diode lasers were used for the detection of these fluorescence proteins/dye : 405nm - mTagBFP2; 488nm - sfGFP; 561nm - sfCherry3C; 640nm - Alexa Fluor 647.
Software	Data was collected using the BD FACSDiva Software and analyzed using the FlowJo software.
Cell population abundance	For each sample, 100,000 cells were analyzed and gated for live, singlet, and then mTagBFP2-positive cells. The number of cells in this gating was dependent on the transfection efficiency for each experiment.
Gating strategy	Live, single, and mTagBFP2 positive cells were gated and were analyzed for sfGFP and sfCherry3C fluorescence.

☒ Tick this box to confirm that a figure exemplifying the gating strategy is provided in the Supplementary Information.
